# Impact of Incident Parkinson's Disease on Satisfaction With Life

**DOI:** 10.3389/fneur.2018.00589

**Published:** 2018-07-25

**Authors:** Elzbieta W. Buczak-Stec, Hans-Helmut König, André Hajek

**Affiliations:** Department of Health Economics and Health Services Research, University Medical Center Hamburg-Eppendorf, Hamburg, Germany

**Keywords:** Parkinson disease, subjective well-being, life satisfaction, longitudinal studies, sex differences

## Abstract

**Aim:** The aim of the study was to investigate the effect of the onset of Parkinson's disease (PD) on life satisfaction.

**Method:** Data from 2008, 2011, and 2014 were used from a population-based prospective cohort (German Ageing Survey; 8,982 observations in FE regression analysis) of community-residing individuals in the second half of life (≥40 years) in Germany. Satisfaction with life was quantified using the established Satisfaction with Life Scale. Physician-diagnosed PD was reported.

**Results:** In total, 48.9% were female. The mean age was 63.8 years (±11.3 years). Average life satisfaction equaled 3.8 (±0.7). Linear fixed effects regressions revealed that the onset of PD was associated with a considerable decline in life satisfaction (β = −0.37, 95% CI −0.69 to −0.05, *p* < 0.05). This effect was significantly more pronounced in men. Moreover, a decrease in life satisfaction was associated with younger age, changes from “employed” to “not employed,” worsening self-rated health, the onset of depression, and an increase in the number of physical illnesses.

**Conclusions:** The onset of PD is associated with a marked reduction of life satisfaction among individuals in the second half of life in the total sample and in men, but not in women. For example, this effect was about twice as large as the effect of depression on life satisfaction. Moreover, the effect of PD on life satisfaction was more pronounced than the effect of a strong decrease in self-rated health (from “very good” to “very bad”) on life satisfaction. Effective treatment of symptoms might contribute to maintaining life satisfaction.

## Introduction

Parkinson's disease (PD) is the second most common neurodegenerative disease. The incidence rises with age. It is characterized by, among others, rigidity, tremor, akinesia, bradykinesia, and/or severe functional limitations. PD is not curable and treatment is limited to symptoms. Consequently, being diagnosed with PD may have a huge impact on individuals' health-related quality of life (HRQoL) and life satisfaction. Whereas HRQoL reflects primarily individuals' health, life satisfaction includes the global assessment of individual's life.

In contrast to the large body of evidence showing an association between PD and decreased HRQoL, only a few *cross-sectional* studies have shown an association of PD with decreased life satisfaction (1–3). Moreover, some of these studies are based on *clinical sample*. Thus, the generalizability to the population level is uncertain. Studies based on a *nationally representative* sample investigating whether the onset of Parkinson influences life satisfaction *longitudinally* are missing. Yet, it is essential to assess life satisfaction in a longitudinal way in order to control for time-constant unobserved heterogeneity (e.g., genetic disposition) (4). Thus, the aim of this study was to analyse the effect of the *onset* of PD on satisfaction with life using a nationally representative prospective cohort of older adults. Enhancing life satisfaction is a main goal of nations because it is associated with morbidity and mortality.

## Materials and methods

### Sample

We used data from German Ageing Survey (DEAS). This large nationwide, longitudinal study of the community-dwelling middle aged and older population in Germany (40 years and over) was launched by the German Federal Government in the mid-1990s. The first wave took place in 1996. Further waves followed in 2002, 2008, 2011, and 2014. In this study, we used data from the 3rd to 5th wave. The DEAS study has a cohort-sequential design introducing additionally new baseline samples every 6 years. The cohort profile is described in further detail elsewhere (5).

Written consent was obtained from all participants. Permission from institutional review boards or ethic committees was not necessary, since the criteria for requiring an ethics statement were not met (e.g., invasive methods). The principles outlined in the Declaration of Helsinki were followed.

### Outcome measure

To assess satisfaction with life we used the well-established Satisfaction with Life Scale (SWLS). SWLS quantifies global life satisfaction based on an individual's unique set of values (6). The scale ranges from 1 to 5, with higher values reflecting higher satisfaction with life.

### Independent variables

PD was assessed according to a list of various illnesses diagnosed by a physician (e.g., incontinence, stroke). The list is based among others on the Charlson Comorbidity Index. The number of individuals who changed their status from “absence of PD” to “presence of PD” are reported in the results section (section Sample characteristics).

Additionally to age, gender, marital status [married, living together with spouse; other (divorced; widowed; single)] we included (log) household net equivalent income and labor force participation (working; retired; other: not employed) as control variables. Moreover, we controlled for physical functioning (7) [subscale of SF-36 Short Form Health Survey (from 0 = worst to 100 = best)], sport frequency (based on the International Physical Activity Questionnaire—daily; several times per week; once a week; 1–3 times a week; less often; never), depression (assessed with the Center for Epidemiological Studies Depression Scale (CES-D) (8); range from 0 to 45; cut-off at ≥18), size of social network (important people in regular contact; from 0 to 9) and self-rated health (from 1 = very good to 5 = very bad). Furthermore, the morbidity was assessed by the total number of physical illnesses (e.g., diabetes) (from 0 to 11).

### Statistical analysis

It is important to control for unobserved factors in life satisfaction research (4). Consequently, linear fixed effects (FE) regressions were used in this study, which is a common method in life satisfaction research. FE regressions provide consistent estimates under weak assumptions. The choice was also supported by the Sargan-Hansen test (Sargan-Hansen statistic was 160.0, *p* < 0.001). The Sargan-Hansen test is a Hausman test with cluster-robust standard errors.

As the aim of this study was to analyze the association between the onset of PD and changes in life satisfaction, we used FE regressions. FE regressions solely exploit changes within individuals over time. For example, intra-individual changes from “absence of PD” to “presence of PD” were used in FE regressions. Thus, only time-varying factors (factors varying within individuals over time such as the onset of high blood pressure) can be included in FE regressions as main effects. However, time-constant factors (factors not varying within individuals over time such as sex) can be included as moderating factors (e.g., sex x PD). Cluster-robust standard errors were computed. The significance level was set at *p* < 0.05. Stata 15.1 (StataCorp, College Station, Texas, USA) was used.

## Results

### Sample characteristics

Table 1 shows the pooled descriptive characteristics of the observations included in linear FE regressions with satisfaction with life as dependent variable. In total, 48.9% were female. The mean age was 63.8 years (±11.3 years). Average life satisfaction equaled 3.8 (±0.7).

**Table 1:** Characteristics of the observations included in linear FE Regressions (Waves 3–5, pooled, *n* = 8,982).

**Variables**	**N (%)/Mean (SD); Range**
Female	4,393 (48.9%)
Age	63.8 (11.3); 40–95
Marital status: Divorced/Widowed/Single (Ref.: married, living together with spouse)	2,496 (27.8%)
Number of important people in regular contact	4.7 (2.8); 0–9
Employment status:	
- Employed	3,081 (34.3%)
- Retired	4,883 (54.4%)
- Other (not employed)	1,018 (11.3%)
Household net equivalent income (in Euro)	1,757.8 (1,589.7);
	156.7–65,000
Frequency of sports activities:	
- Daily	684 (7.6%)
- Several times a week	2,105 (23.4%)
- Once a week	1,584 (17.7%)
−1–3 times a month	676 (7.5%)
- Less often	1,071 (11.9%)
- Never	2,862 (31.9%)
Self-rated health (from 1 = very good to 5 = very bad)	2.5 (0.8); 1–5
Absence of depression (CES-D < 18)	8,469 (94.3%)
Physical functioning (from 0 = worst score to 100 = best score)	83.6 (22.2); 0–100
Absence of Parkinson's disease	8,917 (99.3%)
Number of physical illnesses	2.4 (1.8); 0–10
Life satisfaction	3.8 (0.7); 1–5

*The Satisfaction with Life Scale (SWLS) was used to quantify life satisfaction (6). The Center for Epidemiological Studies Depression Scale (CES-D) was used to quantify depression (8). Physical functioning was measured by the subscale “Physical Functioning” of SF-36 Short Form Health Survey (0–100 range) (7)*.

In total, 22 individuals (12 men, 10 women) changed their status from “absence of PD” to “presence of PD”.

### Regression analysis

Results of FE regression analysis are depicted in Table 2. FE regressions showed that the onset of PD is associated with a decrease in life satisfaction (β = −0.37, *p* < 0.05). Moreover, a decrease in life satisfaction was associated with younger age (β = 0.02, *p* < 0.05), changes from “employed” to “not employed” (β = −0.11, *p* < 0.05), worsening self-rated health (β = −0.05, *p* < 0.01), the onset of depression (β = −0.19, *p* < 0.001), and an increase in the number of physical illnesses (β = −0.04, *p* < 0.001). A decrease in life satisfaction was not associated with the number of important people in regular contact, marital status, the frequency of sports activities, and physical functioning.

**Table 2:** Determinants of life satisfaction. Results of linear FE regression analysis (wave 3 to wave 5).

**Independent variables**	**Total sample ^a^**
Onset of Parkinson's disease	−0.37 ^*^
	(−0.69 to −0.05)
Age	0.02 ^***^
	(0.01 to 0.03)
Marital status (Ref.: married, living together with spouse)	0.11
	(−0.03 to 0.25)
Number of important people in regular contact	0.00
	(−0.01 to 0.01)
Employment status:	
- Retired (Ref.: Employed)	−0.03
	(−0.13 to 0.07)
- Other (not employed)	−0.11 ^*^
	(−0.20 to −0.02)
(Log) household net equivalent income	0.06^+^
	(−0.00 to 0.12)
Frequency of sports activities:	
- Several times a week (Ref.: Daily)	0.01
	(−0.07 to 0.09)
- once a week	0.06
	(−0.03 to 0.15)
−1-3 times a month	0.03
	(−0.07 to 0.12)
- less often	0.04
	(−0.05 to 0.13)
- never	0.03
	(−0.05 to 0.11)
Self-rated health (from 1 = very good to 5 = very bad)	−0.05 ^**^
	(−0.08 to −0.01)
Depression (CES-D ≥ 18)	−0.19 ^***^
	(−0.30 to −0.09)
Physical functioning (from 0 = worst score to 100 = best score)	0.00
	(−0.00 to 0.00)
Number of physical illnesses	−0.04 ^***^
	(−0.05 to −0.02)
Constant	2.09 ^***^
	(1.38 to 2.79)
Observations	8,982
Number of Individuals	6,440
*R*^2^	0.04

a *Beta-coefficients are reported; confidence intervals in parentheses*.

*** p < 0.001,

** p < 0.01,

* *p < 0.05, ^+^p < 0.10. The Satisfaction with Life Scale (SWLS) was used to quantify life satisfaction (6). The Center for Epidemiological Studies Depression Scale (CES-D) was used to quantify depression (8). Physical functioning was measured by the subscale “Physical Functioning” of SF-36 Short Form Health Survey (0–100 range) (7). It is worth noting that the Stata command for FE regression analysis (“xtreg, fe”) include individuals with only one observation in calculating the number of observations because these individuals provide information about the variance components, the constant, the between R^2^ and so on. Nevertheless, it does not affect the beta-coefficients as well as the standard errors*.

In sensitivity analysis, health-related factors (depression, self-rated health, physical functioning, and number of physical illnesses) were excluded from our main model. In terms of significance and effect size, the longitudinal association between PD and life satisfaction remained virtually the same (β = −0.36, *p* < 0.05).

In further sensitivity analysis, regressions were stratified by sex (results not shown, but available upon request). While the onset of PD was associated with a decrease in life satisfaction in men (β = −0.70, *p* < 0.001), the onset of PD was not associated with a decrease in life satisfaction in women (β = −0.05, *p* = 0.83). We further tested whether the longitudinal association between PD and life satisfaction significantly varies by gender (including an interaction term sex x PD). Actually, the interaction term achieved statistical significance (β = −0.63, *p* < 0.05).

## Discussion

Thus far, few studies have shown that PD was associated with decreased life satisfaction cross-sectionally (1–3). Based on a longitudinal study, our findings add to the scarce knowledge based on cross-sectional studies by showing that the *onset* of PD is associated with a decrease in life satisfaction after adjusting for important covariates including mental and physical health. This longitudinal association is conceivable because PD is a critical diagnosis with no possibility to cure. Adjusting for various potential confounders, this longitudinal association might be explained by the association between PD and perceived social exclusion. Moreover, this longitudinal association might be explained by the relation between PD and decreased perceived autonomy (2). In addition, individuals with PD might have low acceptance of illness and may feel helpless due to incurability of PD. It could also be explained by the relation between PD and the increased fear of falling (9). Fear of falling in turn is associated with decreased satisfaction with life (10).

However, more research is required to explore this relation in further detail. Particularly, further research is necessary to investigate potential gender differences, which were observed in our study. Gender differences might be explained by differences in coping strategies between men and women.

This is the first study examining whether the incidence of physician diagnosed PD was associated with a change in life satisfaction longitudinally. A large nationally representative sample of individuals living in private households was used. The widely established SWLS was used to quantify life satisfaction. The problem of unobserved heterogeneity was mitigated using FE regressions.

It might be difficult to generalize our findings to individuals living in institutionalized settings. Another limitation might be that other time-varying unobserved factors (e.g., self-rated financial situation, chronic pain) might affect the relation of interest. Future research is required to clarify this relationship. The number of individuals developing PD was rather low in this population-based sample. However, the incident cases observed in the DEAS study correspond to figures reported from earlier studies (11). Further research is needed based on larger and long-running cohort studies.

## Conclusion

The onset of PD is associated with a marked reduction of life satisfaction among individuals aged 40 and over in the total sample and in men, but not in women. For example, this effect was about twice as large as the effect of depression on life satisfaction. Furthermore, the effect of PD on life satisfaction was more pronounced than the effect of a strong decrease in self-rated health (from “very good” to “very bad”) on life satisfaction. In men, the effect of PD on life satisfaction was about fourfold as large as the effect of depression on life satisfaction. Effective treatment of symptoms might contribute to maintaining life satisfaction.

## Resource identification initiative

Research Data Centre of the German Centre of Gerontology, RRID:SCR_014714).

## Data availability statement

The data used in this study are third-party data. The anonymized data sets of the DEAS (1996, 2002, 2008, 2011, and 2014) are available for secondary analysis. The data has been made available to scientists at universities and research institutes exclusively for scientific purposes. The use of data is subject to written data protection agreements. Microdata of the German Ageing Survey (DEAS) is available free of charge to scientific researchers for non-profitable purposes. The FDZ-DZA provides access and support to scholars interested in using DEAS for their research. However, for reasons of data protection, signing a data distribution contract is required before data can be obtained. Please see for further Information (data distribution contract): https://www.dza.de/en/fdz/access-to-data/formular-deas-en-english.html.

## Author contributions

EBS, HHK and AH: Design and concept of analyses, preparation of data, statistical analysis, and interpretation of data, preparing of the manuscript. All authors critically reviewed the manuscript, provided significant editing of the article and approved the final manuscript.

### Conflict of interest statement

The authors declare that the research was conducted in the absence of any commercial or financial relationships that could be construed as a potential conflict of interest.
